# Awareness of COVID-19 Symptoms, Risk Factors, and Vaccinations in Patients with Multiple Sclerosis

**DOI:** 10.3390/ijerph19063366

**Published:** 2022-03-12

**Authors:** Ewa Krzystanek, Agata Jurczak, Kinga Kocur, Jakub Jurkiewicz, Aleksandra Kaczmarczyk

**Affiliations:** 1 Department of Neurology, Faculty of Health Sciences in Katowice, Medical University of Silesia, 40-635 Katowice, Poland; 2 Students’ Scientific Association, Department of Neurology, Faculty of Medical Sciences in Katowice, Medical University of Silesia, 40-752 Katowice, Poland; agatka.jurczak@gmail.com (A.J.); kingakocur98@wp.pl (K.K.); kubaju97@gmail.com (J.J.); 3 Department of Neurology, Faculty of Medical Sciences in Katowice, Medical University of Silesia, 40-752 Katowice, Poland; akaczmarczyk@sum.edu.pl

**Keywords:** COVID-19, multiple sclerosis, knowledge, vaccination

## Abstract

Multiple Sclerosis (MS) is the most common chronic autoimmune disease of the central nervous system, affecting around 2.8 million people worldwide. Patients’ knowledge about COVID-19 infection, and their proper protective actions, may reduce the risk of infection. The aim of this study was to assess the knowledge of patients with MS about SARS-CoV-2, COVID-19 illness, the relationship between MS and COVID-19, willingness to be vaccinated, and the impact of the pandemic on MS care. An original, anonymous, 35-items, self-reported questionnaire was used in both web-based and on-site survey formats. Two-hundred and forty-eight questionnaires were analyzed (mean age 40.8 ± 10.6 years, 77.8% women). Participants reported the use of multiple sources of information, and the most common were websites (77.8%) and television (59.3%). The majority of participants knew the correct symptoms of COVID-19 or transmission routes (94.4%), and accepted the pandemic’s restrictions (96.8%). A total of 93.2% considered SARS-CoV-2 as highly infectious, and 69% thought they were at higher risk of being infected with SARS-CoV-2, mainly because of immunodeficiency (82.7%). Although most of them were afraid of COVID-19 (69.0%), only two-thirds wanted to be vaccinated. Patients who were afraid of COVID-19 had a 3.5-times higher chance to declare willingness for vaccination. A total of 29.8% patients claimed that the COVID-19 pandemic limited access to the healthcare system. This study shows that Polish patients with MS represent a good level of knowledge about COVID-19 disease, and acceptance for public rules, but their willingness for vaccinations is not sufficient. Country-wide educational campaigns should be conducted, particularly on the internet and TV. Restrictions in healthcare facilities should be balanced to secure access for patients with MS.

## 1. Introduction

Multiple Sclerosis (MS) is the most common chronic autoimmune disease of the central nervous system, affecting around 2.8 million people worldwide, and roughly 42,000 in Poland [1,2,3]. MS is an inflammatory, demyelinating disorder causing axonal degeneration, manifesting with various, disseminated, accumulated neurologic symptoms, including monocular visual loss, double vision, ataxia, sensation or motor deficits, and inevitably leading to disability [1,4]. The clinical course of MS differs between patients, but overall, frequent exacerbations (relapses) may substantially accelerate disease progression.

In Poland, 1725 new cases of MS are diagnosed each year, of which 91% have relapsing-remitting (RR-MS), and 9% have progressive forms, with women being more frequently affected than men (female-to-male ratio, 2.4) [5,6] The median age of patients newly diagnosed with MS is 37 years (IQR 28-48) at their first diagnosis, whereas the median age of the overall population of MS patients in Poland is 50 years (interquartile range, IQR, 39–61) [5,6], which is in line with the global population. The median time from the first symptoms to MS diagnosis is 7.4 months, and from MS diagnosis to treatment initiation is 18.48 months [7]. The greatest challenge for both clinicians and patients is the disability progression, which limits the patients in various aspects, and worsens their quality of life [8].

Patients with MS have a higher risk of comorbidities, either physical or mental conditions, as compared with the general population [9,10,11]. The presence of MS increases the risk of infections, more severe infections, and a higher need for intensive care [12,13,14]. Moreover, patients treated with newer disease-modifying treatments (DMTs), which exert stronger effects on the immune defense, may have a higher risk of certain infections [15,16]. Recurring infections in patients with MS are of particular concern for clinicians, as these may impact the clinical presentation of MS, and have implications for treatment choice, adherence, and outcome. The occurrence of relapses is more frequent after viral infections, and the incidence of relapses correlates with the temporal distribution of infections [17]. Infections are also the main cause of death in MS [13,18].

By the end of year 2019, a novel coronavirus disease (abbreviated as COVID-19) caused by the severe acute respiratory syndrome coronavirus 2 (SARS-CoV-2) has spread worldwide. WHO declared COVID-19 a pandemic in March 2020, and until December 2021, over 5,440,000 deaths due to COVID-19 were reported [19]. SARS-CoV-2 infection is clinically manifested with pulmonary, gastrointestinal, skin, or neurological symptoms [20,21]. Since the virus is transmitted mainly via droplets, specific practices are recommended for routine infection prevention during the COVID-19 pandemic, such as: use of personal protective equipment (masks, gloves), frequent sanitization, and physical distancing [22]. Although studies showed that MS is not a risk factor for a severe COVID-19 course, patients’ knowledge of possible risks and their actions may help in reducing the risk of infection.

Reports on the general status of knowledge about the COVID-19 pandemic in Poland are very limited. In a recently published nationwide online survey with 1002 participants, the overall rate of correct answers to specific questions about COVID-19 ranged from 44.6% to 84.1%, and the average was 60.1%, with the lower level of knowledge about the COVID-19 pandemic being associated with a greater severity of psychopathological symptoms [23]. Willingness for vaccinations against SARS-CoV-2, or their hesitancy, is most often considered as a surrogate for an adequate level of knowledge; however, published studies are limited to narrow samples, rarely going beyond a single country; therefore, they may have a limited ability to adequately ascertain a general level of knowledge, or to explain the differences between populations [24]. Moreover, the level of knowledge of people having any connection with the medical field (e.g., healthcare workers or medical students) may be different from those of other professions. For example, Polish healthcare system workers significantly more often demonstrated their willingness to get vaccinated against SARS-CoV-2 when compared to the control group drawn from the general population (82.95% vs. 54.31%, respectively) [25]. Similarly, more Polish medical students indicated a will to get vaccinated than non-medical students (91.99% vs. 59.42%) [26]. In Poland, COVID-19 vaccine hesitancy is quite high [27], with more than 30% still unwilling to get vaccinated [28].

Prevention of infections is a key in the multidisciplinary management of patients with MS, and vaccinations are one of the major long-term strategies [29]. However, vaccination willingness and patients’ hesitancy may determine the possibility of the application of such protection at individual and population levels [30,31]. Data on specific knowledge of patients with different diseases about COVID-19 and/or their willingness for vaccinations are not available in Poland. Admittedly, adequate knowledge of patients about vaccinations is essential to achieve an appropriate level of prevention [27,32].

It is not known whether MS patients have sufficient knowledge about COVID-19 disease to act responsibly. Therefore, the aim of this study was to assess the knowledge of patients with MS about SARS-CoV-2, COVID-19 illness, their perception of the possible relationship between MS and COVID-19, their willingness to be vaccinated (and reasons for this decision), and, additionally, gather observations about the possible impact of the pandemic on MS care.

## 2. Materials and Methods

The study was carried out in both web-based and on-site survey formats. Microsoft Office Forms, online forms designed for electronic questionnaires, were distributed among several MS Patients’ Advocacy Groups (PAG; the list of PAGs participating is provided in the Acknowledgments section below). Patients associated with PAGs were instructed how to fill in an online form, and submitted their responses electronically. Moreover, paper-based forms were distributed among MS patients visiting the Department of Neurology and outpatient clinic in the tertiary hospital located in the main city of the province. Patients invited to the study were adults, with an established diagnosis of MS, and who were willing to provide the responses to the questionnaire. Participants who completed the survey online constituted an ‘online group’, and participants who completed the survey during their visits constituted an ‘on-site group’. The study was conducted from January 2021 to March 2021. Participants were not compensated for completing the survey.

An original, anonymous, 35-items, self-reported questionnaire was used in the study. It consisted of 24 closed-ended questions, 4 open-ended questions, and 7 multiple choice questions, including the “Other” response, allowing for additional explanations. The questionnaire aimed to collect the participant’s knowledge about COVID-19 illness, and their opinion about vaccinations and the possible pandemic impact on individual or general MS management.

In the first part, the questionnaire queried demographic (sex, age, level of education, profession, and place of residence, e.g., rural vs. urban) and basic clinical data (MS subtype, onset, duration, major symptoms, current MS medication, and comorbidities). MS severity was captured as a self-reported disability, and graded according to the Expanded Disability Status Scale (EDSS) [33]: 1. no disability, minimal signs of MS; 2. symptoms present with minimal disability; 3. moderate disability, but no restrictions to ambulation; 4. significant disability, but independent for most of the day, able to walk without aid or rest for about 500 m; 5. disability severe enough to impair full daily activities, able to walk without aid or rest for about 200 m; 6. unilateral or bilateral cane or crutch required to walk for about 100 m with or without resting; 7. unable to walk, essentially restricted to wheelchair. Currently received MS medications reported by participants were categorized according to presently used Disease-Modifying Treatment (DMT) drug class categories.

The following parts of the questionnaire consisted of questions pertaining to methods and frequency of acquisition of the information about COVID-19 illness and/or SARS-CoV-2, main information sources, perception and understanding of official pandemic restrictions, participants’ opinion on possible interrelation of MS and SARS-CoV-2 infection, and their opinion on vaccinations. Patients were also asked whether they already had a COVID-19 infection. Clinical severity of COVID-19 illness, if present, was graded based on the reported course, such as: asymptomatic or only isolated loss of smell and/or taste, mild symptoms reported as influenza-like illness (fatigue, cough, fever), moderate course leading to limitation in functioning, severe state requiring hospitalization and high-flow oxygenation, and a critical state requiring mechanical ventilation and management in an intensive care unit (ICU). Finally, participants were asked their opinion about the COVID-19 pandemic’s impact on MS therapy, and whether it affected healthcare system accessibility for patients with MS. For further analysis, answers to the open-ended questions, e.g., level of disability, MS medication, or COVID-19 illness severity were categorized as described above.

Data were presented as a mean (±standard deviation, SD), median (min–max), or percentages (*n* (%)), with percentages rounded to one decimal where applicable. Between-group comparisons were performed using a chi-squared test, Fisher’s exact test, t-test, or Mann–Whitney U test with STATISTICA 13 PL software (TIBCO Statistica, v. 13.3.0, TIBCO Software Inc., Palo Alto, CA, USA). To quantify associations of demographic and baseline characteristics with responses about patients’ knowledge, we used a regression analysis. Primarily, we assessed the association between parameters using correlation analysis. After determining the strongest correlations, we used stepwise regression model selection, and for binary independent variables, we used logistic regression analysis. *p*-values are intended only as descriptive measures, and should be interpreted with caution. A *p*-value below 0.05 was considered significant.

## 3. Results

A total of 292 surveys were collected, 164 were completed on-site, and 128 were submitted online. A total 248 questionnaires were accepted (134 (54.0%) on-site, and 114 (46.0%) online), and 44 were excluded due to incomplete or missing information. The mean age of included participants was 40.8 (±10.6) years, and the majority of participants were women (77.8%), and most often (92.7%) reported at least 10 years of education, and were white-collar workers (54.4%). Patients predominantly reported the relapsing-remitting form of MS (RR-MS) (82.7%), with a median 9 years duration of the disease; however, a slightly shorter duration was reported by men (median 8 years) than in women (*p* = 0.046). At the time of the survey, 83.5% were taking DMT, with dimethyl fumarate being the most frequently used (31.5%), and 43.1% suffered from comorbidities. Detailed demographics and clinical characteristics are presented in Table 1.

### 3.1. Knowledge about COVID-19

The majority of respondents (95.2%) reported a constant search for the most recent information about COVID-19 disease, with significantly more women being active in information searches than men (96.9% vs. 89.1%, *p* = 0.028). Moreover, retired persons (*p* = 0.001) and patients in the ‘stationary group’ (*p* = 0.005) searched for information about the COVID-19 pandemic more frequently than the others. Overall, 52.4% patients did it at least once a day, and 4.4% less than once a week, with the internet (including social media) and television being the most frequently used sources of information. As the questionnaire was extensive, and it was not possible to include all the data in one table, only responses provided to selected questions are given in Table 2, with the rest of results provided in the following subsections.

**Figure 1:**
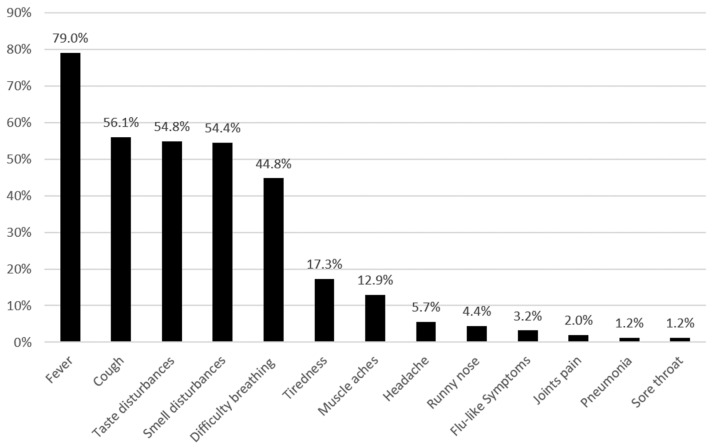
The most common symptoms recognized by patients with MS as indicative of COVID-19 illness (provided for the frequency greater than or equal to 1%).

The majority of respondents (182, 73.4%) were aware of the current phase of the COVID-19 pandemic. Moreover, 202 patients (81.5%) knew the proper name of the virus (SARS-CoV-2). Among ways of the virus’ transmission, the most frequently reported were airborne and direct contact (94.4% and 58.1%, respectively). The most frequent symptoms reported as indicative of COVID-19 illness were: fever, cough, taste and smell disturbances, and difficulties with breathing (Figure 1).

SARS-CoV-2 was considered as highly infectious by 231 (93.2%) of respondents, and 236 (95.2%) agreed that children can also be infected. Most participants (240; 96.8%) claimed to follow the pandemic’s restrictions. However, 97 (39.1%) thought that shutting down public places does not have a relevant effect on spreading a virus. Furthermore, 203 (81.9%) claimed that the epidemiological situation in Poland is no worse than in other countries, and 218 (87.9%) were aware that the quarantine duration in Poland is 10 days.

Most of the respondents (171; 69.0%) were afraid of being infected with SARS-CoV-2. A total of 150 (60.5%) participants claimed that, due to MS, they are at a higher risk of being infected with SARS-CoV-2. Among those, 82.7% identified immunodeficiency during MS as the most common reason for a higher risk of infection, with an additional 46.0% and 34.0% of responses, respectively, pointing to chronic disease (MS) and necessity of frequent visits in healthcare facilities in patients with MS being important factors increasing the risk of infection (Table 2). Women (64.2% vs. 47.3%; OR = 2.01; 95% CI: 1.09, 3.67; *p* = 0.024) and patients in the online group (70.2% vs. 52.2%; OR = 2.15; 95% CI: 1.27, 3.64; *p* = 0.004) more frequently claimed that MS patients are more prone to COVID-19 infection than their counterparts. Moreover, patients with restricted ambulation (disability score 4–7) more frequently claimed that MS patients are more prone to COVID-19 infection (74.3% vs. 55.1%; *p* = 0.002), and disability score was found to be an independent predictor of such an opinion (OR = 1.21; 95% CI: 1.05, 1.40; *p* = 0.007).

### 3.2. COVID-19 Infection and Vaccinations

Up to Spring 2021, only 19% of respondents reported a previous infection with SARS-CoV-2, with a numerically higher proportion of men than women (23.6% vs. 17.6%). In majority of patients with COVID-19 illness, the disease course was of mild or moderate severity, with only three women (6.4% infected patients) requiring hospitalization. An asymptomatic course of COVID-19 disease was reported by 12 respondents (25.5% infected), mild symptoms were reported by 12 patients (25.5% infected), and a moderate course occurred in 20 respondents (42.6% infected).

Willingness for vaccination against SARS-CoV-2 was declared only by around 60% of respondents. A belief that the vaccine is not sufficiently studied, and concerns of adverse side-effects, were the major reasons for vaccination rejection (Table 2). Patients who were afraid of COVID-19 infection had a 3.5-times higher chance to declare willingness for vaccination than those who were not afraid (69.0% vs. 39.0%; OR = 3.49; 95% CI: 1.99, 6.11; *p* < 0.001). Age, sex, education level, profession, place of residence, type of MS, level of disability, presence of comorbidities, MS phenotype, or SARS-CoV-2 infection in the past were not associated with a greater willingness for vaccination.

### 3.3. MS Care during COVID-19 Pandemic

The majority of participants (70.2%) did not share the opinion that the impact of the COVID-19 pandemic resulted in limited access to the healthcare system; however, significantly more women (33.2% vs. 18.2%, *p* = 0.032) complained about worsened MS care during the pandemic. Almost one-third of respondents raised the concern that the COVID-19 pandemic has had a negative impact on medical care, and of those, 83.8% complained about more difficult access to physicians, and 25.7% about shorter appointments. Moreover, 14.9% of patients complained about increased controlling procedures upon entering the hospital, and 14.9% had a willingness to reduce contacts with other people, including doctors (Table 2). Participants in the online group significantly more frequently claimed that MS care worsened during the pandemic than participants in the on-site group (49.1% vs. 13.4%; OR = 6.22; 95% CI: 3.36, 11.54; *p* < 0.001).

## 4. Discussion

During the COVID-19 pandemic, the identification of people’s attitudes is highly important, as it can show the extent to which people are reacting differently to the crisis, and help us to understand why some people are more likely than others to follow guidelines aimed at limiting the spread of the virus [34]. To our knowledge, this is the first study evaluating the level of knowledge about COVID-19 and SARS-CoV-2, and attitudes towards vaccines, in Polish patients with MS.

### 4.1. Knowledge about COVID-19

Our study shows that Polish MS patients show a good level of knowledge about COVID-19 disease. Studied participants were able to list most common initial symptoms of COVID-19 (fever, cough), the virus’ transmission routes, and named the virus correctly, which is in line with other reports [20,21]. They were also aware of the high transmission capacity of SARS-CoV-2. Surprisingly, most of them rejected the statement that shutting down public places as a result of pandemic restriction has a relevant impact on SARS-CoV-2 spreading. According to Khataee et al., social distancing might reduce basic reproduction number of the infection [35]. Moreover, it was found that the most commonly spread piece of misinformation concerns policies of managing the pandemic [36]. Despite participants’ awareness of COVID-19, the necessity of highlighting the rules of keeping distances is required for better pandemic management. Comparing our results to Sahraian et al.’s survey, Iranians more often correctly recognized the actual phase of the COVID-19 pandemic, and almost all of them claimed that shutting down crowded places is helpful in limiting the spread of the disease [37]. However, our participants more often stated that children can be infected with COVID-19. These differences may come from sociocultural factors, and an earlier time for the Iranian study.

In the presented study, participants most commonly used websites other than social media to collect information about the pandemic, followed by television, whereas in Indonesia, social media (Facebook, Instagram) was considered the primary source of information [38]. These findings indicate that an internet-based information campaign might be the most effective.

### 4.2. Willingness to Get Vaccination against COVID-19

After one-and-a-half years of the pandemic duration, anti-SARS-CoV-2 vaccination is the only available long-term strategy for the pandemic’s management, and infection prevention. According to the National MS Society, the mRNA COVID-19 vaccines (Pfizer BioNTech and Moderna) or the vector vaccine (J&J) are recommended for MS patients, especially for those with a higher risk of hospitalization (e.g., older, with progressive type of MS, higher disability level, and comorbidities) [39]. However, vaccine hesitancy influenced by patients’ perception of new vaccines as less safe than older vaccines is quite common, and may curb the pandemic’s management [30,31]. Moreover, our study was conducted at the very beginning of the country-wide systemic vaccination program application; therefore, vaccine hesitancy could reflect a low level of knowledge about new vaccines.

The World Health Organization (WHO) and the Centers for Disease Control and Prevention (CDC) recommend COVID-19 vaccination as a game-changing strategy to end the COVID-19 pandemic [40,41]. In Poland, the distribution of vaccines begun on 27 December 2020 [42], and the first recommendations by the Polish Neurological Society about vaccination against COVID-19 for MS patients appeared online in January 2021 [43], and were published in February 2021 [44].

COVID-19 vaccine hesitancy was slightly higher in our study (40.3%) compared to the general population in Poland (31.3%) [45]. According to Raciborski et al., in Poland, the lack of a willingness to be vaccinated was caused generally by concerns about the potential side-effects and the lack of effectiveness of the COVID-19 vaccine. Higher vaccine acceptance in the general adult population in Poland was associated with older age. In addition, among respondents older than 65 years, males declared a greater willingness to get vaccinated [45]. A higher prevalence of women in our study compared to the population studied by Raciborski et al. may explain this difference.

Interestingly, in other countries, willingness to be vaccinated among patients with MS is significantly higher [46,47]. In a study conducted in Portugal from December 2020 to January 2021, and with a similar number of respondents, about 81% of patients were willing to receive a vaccine against SARS-CoV-2. A greater willingness to get vaccinated was associated with demographic factors, such as age, additional comorbidities, and, moreover, with previous vaccination practices and concerns about COVID-19 [46]. In our study, none of the demographic factors were associated with a higher acceptance for vaccinations, which, in general, is in line with the US-based study, except for a higher level of education reported to be associated with a greater willingness to receive the vaccine [47]. However, we had to select an arbitrary cut-off in our study to generally differentiate basic from mid/higher education levels, as there have been several changes to the educational system in Poland throughout the last 20 years. Moreover, only around 7% of our participants had a basic level of education; thus, this may not be fully representative, and direct comparisons are not possible.

It was found that Poles who were afraid of the COVID-19 vaccine were mainly concerned with the severity of side-effects, and also, unknown long-term effects and serious allergic reactions. They also believed that the vaccine does not protect against the infection, or that the effects of vaccination could be temporary [48]. COVID-19 vaccine hesitancy in a Portuguese MS population was associated with potential adverse side-effects, and the vaccine’s safety profile. Patients who were less concerned about COVID-19 disease reported poor interest in vaccination [46]. According to a US follow-up study, fear of long-term potential effects, and doubt in the vaccine approval process were associated with vaccine hesitancy [49]. Respondents in our study showing a poor willingness to be vaccinated against SARS-CoV-2 explained their hesitancy with similar factors: fear of adverse side-effects, and the belief that the vaccine has not been sufficiently studied, and that it is neither beneficial nor necessary. On the other hand, similar to Ehde et al. [47], patients who were afraid of COVID-19 infection had a 3.5-times higher chance to declare a willingness for vaccination than those who were not afraid, which may be useful in future educational campaigns.

### 4.3. MS and COVID-19 Interrelation and Pandemic’s Influence on Healthcare System

Nearly 2/3 of participants claimed that MS patients are more prone to COVID-19 infection. The main reported reasons were immunodeficiency, presence of chronic disease, and the belief that hospital visits increase infection risk. In a US-based survey, about 70% of respondents emphasized an increased risk of COVID-19 infection because of MS [50]. According to Louapre et al., age, higher disability level, and obesity were independent risk factors of COVID-19 [51]. The results of initial observational studies are not consistent or conclusive regarding whether MS patients are at greater risk of COVID-19 infection or not [52,53]. In our study, the overall hospitalization rate due to COVID-19 was 6.4%, which is similar to the Polish cohort of MS patients (6.81%) reported by Czarnowska et al. [54], and comparable to the general Polish population (7.98%) [55].

The pandemic has had a major impact on patients’ everyday functioning [51,56]. As an infectious factor with high morbidity and mortality, SARS-CoV-2 caused a series of restrictions on public life to prevent the virus transmission. Limits were also put on doctor’s appointments and rehabilitation programs, causing difficulties in patient activation programs, and in regular contacts with medical care [57]. Since MS patients are treated with immunosuppressants (DMT), at the pandemic’s outbreak, the Multiple Sclerosis International Federation recommended to balance the risk of possible COVID-19 complications due to MS medications with the risk of stopping MS treatment. Hence, the delay of some DMTs was initially recommended by some of the national MS societies [58,59].

Nearly 1/3 of our participants claimed that their MS care worsened during the COVID-19 pandemic. This statement was associated with the female gender, and being in the ‘online group’. Two of the most mentioned reasons were difficult access to the specialist, and shortened appointments. US studies showed that, in some cases, the pandemic was the main reason for changes in DMTs. Moreover, a significant number of respondents postponed or cancelled scheduled medical visits, laboratory tests, or MRI evaluations because of COVID-19 disease [50,60], whereas according to Zhang et al., the majority of them made these decisions on their own [50]. Therefore, it is necessary to explain to patients that despite the pandemic, the continuation of MS care is essential.

### 4.4. Study Limitations

Our study group may not be representative of the whole MS population in Poland, as a substantial number of questionnaires were collected online, which may be difficult for older persons and those who are less tech-savvy. Moreover, patients invited by PAGs were from different regions of Poland, and patients providing their responses on-site were from one MS center only. As is the nature of surveys, participation in the study required active volunteering; thus, patients not associated with PAGs, those staying at home, or those with less frequent medical visits could miss the opportunity to provide the answers.

A self-reported questionnaire was used in our study, which might include recall bias. We also assessed vaccination willingness and COVID-19 awareness in different pandemic phases, which may differ depending on the pandemic’s waves. Moreover, as knowledge about COVID-19 is rapidly changing, it is probable to misjudge participants’ awareness. Considering these limitations, further research is needed.

## 5. Conclusions

The majority of patients with MS know the symptoms of COVID-19, and almost all of them accept new public rules. Although most of them are afraid of COVID-19, only 60% want to be vaccinated. One-third of patients complained about worsened MS care due to the pandemic.

The main source of information about SARS-CoV-2 and the COVID-19 pandemic is TV and the internet; thus, it is tempting to focus on the educational campaigns conducted, particularly in the electronic media, to reach the widest pool of patients. However, this approach may exclude individuals not familiar with the technology. Individual discussions aimed at vaccinations and the special requirements of MS patients are necessary, and may involve physicians and nurses. Operational restrictions in healthcare facilities caused by the COVID-19 pandemic should be properly balanced to secure comfortable access for patients with MS.

## Figures and Tables

**Table 1:** Demographic and clinical characteristics of all respondents with multiple sclerosis, and differences between men and women.

Characteristic		All Patients	Men	Women	*p*-Value
		*n* = 248	*n* = 55	*n* = 193	
Age (years)	Mean (SD)	40.8 (10.6)	39.7 (12.0)	41.2 (10.2)	0.353 ^†^
Education level	≥10 years	230 (92.7)	48 (87.3)	182 (94.3)	0.084 **
	<10 years	18 (7.3)	7 (12.7)	11 (5.7)	
Profession	White-collar worker	135 (54.4)	23 (41.8)	112 (58.0)	0.084 *
	Retired	67 (27.0)	17 (30.9)	50 (25.9)	
	Blue-collar worker	29 (11.7)	11 (20)	18 (9.3)	
	Unemployed	17 (6.9)	4 (7.3)	13 (6.7)	
Place of residence	Big town (≥100,000 inhabitants)	134 (54.1)	29 (52.7)	105 (54.4)	0.283 *
	Small town (<100,000 inhabitants)	73 (29.4)	20 (36.4)	53 (27.5)	
	Rural area	41 (16.5)	6 (10.9)	35 (18.1)	
Phenotype of Multiple Sclerosis	Relapsing-Remitting	205 (82.7)	46 (83.6)	159 (82.4)	0.816 *
	Secondary Progressive	23 (9.3)	4 (7.3)	19 (9.8)	
	Primary Progressive	20 (8.0)	5 (9.1)	15 (7.8)	
Time since diagnosis	Median (min–max)	9 (1–43)	8 (1–27)	9 (1–43)	0.046 ^‡^
Self-reported disability score ^a^	Median (min–max)	2 (1–7)	2 (1–7)	2 (1–7)	0.515 ^‡^
Disease-Modifying Therapy	No treatment	41 (16.5)	5 (9.1)	36 (18.7)	0.290 *
	Dimethyl fumarate	78 (31.5)	21 (38.2)	57 (29.5)	
	Interferons	34 (13.7)	8 (14.5)	26 (13.5)	
	Glatiramer acetate	23 (9.3)	5 (9.1)	18 (9.3)	
	Ocrelizumab	19 (7.7)	6 (10.9)	13 (6.7)	
	Fingolimod	17 (6.9)	4 (7.3)	13 (6.7)	
	Teriflunomide	17 (6.9)	1 (1.8)	16 (8.3)	
	Natalizumab	15 (6.0)	3 (5.5)	12 (6.2)	
	Cladribine	3 (1.2)	1 (1.8)	2 (1.0)	
	Alemtuzumab	1 (0.4)	1 (1.8)	0	
Comorbidities	Yes	107 (43.1)	18 (32.7)	89 (46.1)	0.077 *
	No	141 (56.9)	37 (67.3)	104 (53.9)	
Comorbidities reported ^b^	Hypothyroidism	38 (15.3)	1 (1.9)	37 (19.2)	
	Hypertension	29 (11.2)	10 (18.2)	19 (9.8)	
	Depression	18 (7.3)	3 (5.5)	15 (7.8)	
	Diabetes	11 (4.4)	5 (9.0)	6 (3.1)	
	Asthma	8 (3.2)	3 (5.5)	5 (2.6)	
	Epilepsy	5 (2.0)	0	5 (2.6)	
	Other	37 (15.0)	3 (5.5)	34 (17.6)	

Note: Unless otherwise indicated, presented data are numbers and percentages of patients within the reported category. ^a^ Self-reported disability scores: 1. no disability, minimal signs of MS; 2. symptoms present with minimal disability; 3. moderate disability, but ambulation not restricted; 4. significant disability, but independent for most of a day, able to walk without aid or rest for about 500 m; 5. disability severe enough to impair full daily activities, able to walk without aid or rest for about 200 m; 6. unilateral or bilateral cane or crutch required to walk for about 100 m with or without resting; 7. Unable to walk, essentially restricted to wheelchair. ^b^ Patients could report more than one comorbidity. Statistics: * chi-squared test, ** Fisher’s exact test, ^†^ t-test, ^‡^ Mann–Whitney U test. Differences in subcategories not analyzed; thus, *p*-values not provided.

**Table 2:** Status of knowledge about COVID-19, SARS-CoV-2 infection, and vaccination willingness among MS patients, men and women.

Characteristic		All Patients	Men	Women	*p*-Value
		*n* = 248	*n* = 55	*n* = 193	
Searching for most updated information on COVID-19	Yes	236 (95.2)	49 (89.1)	187 (96.9)	0.028 **
	No	12 (4.8)	6 (10.9)	6 (3.1)	
Frequency of information updates ^b^	A few times a day	23 (9.3)	5 (9.1)	18 (9.3)	0.043 ^‡^
	Once a day	107 (43.1)	29 (52.7)	78 (40.4)	
	Once a few days	72 (29.0)	12 (21.8)	60 (31.1)	
	Once a week	17 (6.9)	2 (3.6)	15 (7.8)	
	Less than once a week	11 (4.4)	0	11 (5.7)	
	I’m not interested	6 (2.4)	1 (1.8)	5 (2.6)	
Sources of information about COVID-19 ^a^	Websites	193 (77.8)	40 (72.7)	153 (79.3)	
	Television	147 (59.3)	32 (58.2)	115 (59.6)	
	Social media	66 (26.6)	18 (32.7)	48 (24.9)	
	Radio	58 (23.4)	10 (18.2)	48 (24.9)	
	Friends/Family	36 (14.5)	8 (14.5)	28 (14.5)	
	Press	35 (14.1)	5 (9.1)	30 (15.5)	
	Other	7 (2.8)	2 (3.6)	5 (2.6)	
Knowledge on infection transmission ^a^	Airborne	234 (94.4)	54 (98.2)	180 (93.3)	0.727 *
	Direct contact	144 (58.1)	34 (61.8)	110 (57)	
	Oral	24 (9.7)	8 (14.5)	16 (8.3)	
	Sex-transmitted	44 (17.7)	10 (18.2)	34 (17.6)	
Are MS patients more prone to COVID-19?	Yes	150 (60.5)	26 (47.3)	124 (64.2)	0.024 *
	No	98 (39.5)	29 (52.7)	69 (35.8)	
Why? ^a,b^	Immunodeficiency	124 (82.7)	23 (88.5)	101 (81.5)	
	Each disease worsens prognosis	69 (46.0)	10 (38.5)	59 (47.6)	
	Medical appointments increase the risk of infection	51 (34.0)	12 (46.2)	39 (31.5)	
	DMT worsen prognosis	24 (16.0)	5 (19.2)	19 (15.3)	
	other	4 (2.7)	1 (3.8)	3 (2.4)	
Do you want to be vaccinated?	Yes	148 (59.7)	31 (56.4)	117 (60.6)	0.570 *
	No	100 (40.3)	24 (43.6)	76 (39.4)	
Why not? ^a,b^	Vaccine is not sufficiently studied	62 (62.0)	13 (54.2)	49 (64.5)	
	Possible adverse side-effects	58 (58.0)	13 (54.2)	45 (59.2)	
	Is unnecessary	12 (12.0)	6 (25.0)	6 (7.9)	
	It doesn’t work	5 (5.0)	2 (8.3)	3 (3.9)	
	other	19 (19.0)	3 (12.5)	16 (21.1)	
Had COVID-19 in the past?	Yes	47 (19.0)	13 (23.6)	34 (17.6)	0.315 *
	No	201 (81.0)	42 (76.4)	159 (82.4)	
Severity of COVID-19 illness ^b^	Asymptomatic	12 (25.5)	2 (15.4)	10 (29.4)	
	Mild	12 (25.5)	8 (61.5)	4 (11.8)	
	Moderate	20 (42.6)	3 (23.1)	17 (50)	
	Hospitalization	3 (6.4)	0	3 (8.8)	
	Mechanical ventilation	0	0	0	
MS care worsened during COVID-19 pandemic	Yes	74 (29.8)	10 (18.2)	64 (33.2)	0.032 *
	No	174 (70.2)	45 (81.8)	129 (66.8)	
Why? ^a,b^	Harder to get to a doctor	62 (83.8)	7 (70.0)	55 (86.0)	
	Shorter appointments	19 (25.7)	1 (10.0)	18 (28.1)	
	Screening test before entering	11 (14.9)	1 (10.0)	10 (15.6)	
	Patient cancels appointment to avoid contact with other persons	11 (14.9)	2 (20.0)	9 (14.1)	
	other	13 (17.6)	1 (10.0)	12 (18.8)	

Note: Presented data are numbers and proportions of patients within the reported category. ^a^ Patients could provide more than one answer in the respective category. ^b^ Percentages provided only for patients responding in that category. Statistics: * chi-squared test, ** Fisher’s exact test, ^‡^ Mann–Whitney U test. MS, multiple sclerosis; DMT, disease-modifying therapy. Differences in subcategories not analyzed; thus, *p*-values not provided.

## Data Availability

The data used to support the findings of this study are included within the article.

## References

[1] Reich D.S., Lucchinetti C.F., Calabresi P.A. (2018). Multiple Sclerosis. N. Engl. J. Med..

[2] Walton C., King R., Rechtman L., Kaye W., Leray E., Marrie R.A., Robertson N., La Rocca N., Uitdehaag B., van der Mei I. (2020). Rising prevalence of multiple sclerosis worldwide: Insights from the Atlas of MS, third edition. Mult. Scler. J..

[3] Kapica-Topczewska K., Brola W., Fudala M., Tarasiuk J., Chorazy M., Snarska K., Kochanowicz J., Kulakowska A. (2018). Prevalence of multiple sclerosis in Poland. Mult. Scler. Relat. Disord..

[4] Lad S.P., Chapman C.H., Vaninetti M., Steinman L., Green A., Boakye M. (2010). Socioeconomic trends in hospitalization for multiple sclerosis. Neuroepidemiology.

[5] Wnuk M., Maluchnik M., Perwieniec J., Podwojcic K., Szelag M., Walkiewicz D., Zakrzewski M., Kulakowska A., Brola W., Rejdak K. (2021). Multiple sclerosis incidence and prevalence in Poland: Data from administrative health claims. Mult. Scler. Relat. Disord..

[6] Atlas of MS. https://www.atlasofms.org/fact-sheet/poland.

[7] Kapica-Topczewska K., Collin F., Tarasiuk J., Chorąży M., Czarnowska A., Kwaśniewski M., Brola W., Bartosik-Psujek H., Adamczyk-Sowa M., Kochanowicz J. (2020). Clinical and epidemiological characteristics of multiple sclerosis patients receiving disease-modifying treatment in Poland. Neurol. I Neurochir Pol..

[8] Brola W., Sobolewski P., Fudala M., Flaga S., Jantarski K., Ryglewicz D., Potemkowski A. (2016). Self-reported quality of life in multiple sclerosis patients: Preliminary results based on the Polish MS Registry. Patient Prefer. Adherence.

[9] Chou I.J., Kuo C.F., Tanasescu R., Tench C.R., Tiley C.G., Constantinescu C.S., Whitehouse W.P. (2020). Comorbidity in multiple sclerosis: Its temporal relationships with disease onset and dose effect on mortality. Eur. J. Neurol..

[10] Marrie R.A. (2017). Comorbidity in multiple sclerosis: Implications for patient care. Nat. Rev. Neurol..

[11] Persson R., Lee S., Ulcickas Yood M., Wagner C.M., Minton N., Niemcryk S., Lindholm A., Evans A.M., Jick S.S. (2020). Infections in patients diagnosed with multiple sclerosis: A multi-database study. Mult. Scler. Relat. Disord..

[12] Marrie R.A., Elliott L., Marriott J., Cossoy M., Blanchard J., Tennakoon A., Yu N. (2014). Dramatically changing rates and reasons for hospitalization in multiple sclerosis. Neurology.

[13] Montgomery S., Hillert J., Bahmanyar S. (2013). Hospital admission due to infections in multiple sclerosis patients. Eur. J. Neurol..

[14] Wijnands J.M., Kingwell E., Zhu F., Zhao Y., Fisk J.D., Evans C., Marrie R.A. (2017). Tremlett, H. Infection-related health care utilization among people with and without multiple sclerosis. Mult. Scler..

[15] Brück W., Gold R., Lund B.T., Oreja-Guevara C., Prat A., Spencer C.M., Steinman L., Tintoré M., Vollmer T.L., Weber M.S. (2013). Therapeutic decisions in multiple sclerosis: Moving beyond efficacy. JAMA Neurol..

[16] Luna G., Alping P., Burman J., Fink K., Fogdell-Hahn A., Gunnarsson M., Hillert J., Langer-Gould A., Lycke J., Nilsson P. (2020). Infection risks among patients with multiple sclerosis treated with fingolimod, natalizumab, rituximab, and injectable therapies. JAMA Neurol..

[17] Andersen O., Lygner P.E., Bergström T., Andersson M., Vahlne A. (1993). Viral infections trigger multiple sclerosis relapses: A prospective seroepidemiological study. J. Neurol..

[18] Smestad C., Sandvik L., Celius E.G. (2009). Excess mortality and cause of death in a cohort of Norwegian multiple sclerosis patients. Mult. Scler..

[19] World Health Organization WHO Coronavirus (COVID-19) Dashboard. https://covid19.who.int/.

[20] Mohamadian M., Chiti H., Shoghli A., Biglari S., Parsamanesh N., Esmaeilzadeh A. (2021). COVID-19: Virology, biology and novel laboratory diagnosis. J. Gene Med..

[21] Salian V.S., Wright J.A., Vedell P.T., Nair S., Li C., Kandimalla M., Tang X., Carmona Porquera E.M., Kalari K.R., Kandimalla K.K. (2021). COVID-19 Transmission, Current Treatment, and Future Therapeutic Strategies. Mol. Pharm..

[22] Centers for Disease Control and Prevention Interim Infection Prevention and Control Recommendations for Healthcare Personnel During the Coronavirus Disease 2019 (COVID-19) Pandemic. https://www.cdc.gov/coronavirus/2019-ncov/hcp/infection-control-recommendations.html.

[23] Maciaszek J., Lenart M., Misiak B., Grzebieluch J., Gawłowski P., Ciułkowicz M., Łuc D., Szcześniak D., Rymaszewska J. (2021). Unknown Enemy and Psychopathological Responses: A Cross-Sectional Nationwide Study Assessing the Knowledge about COVID-19. Front. Psychiatry.

[24] Walkowiak M.P., Walkowiak J.B., Walkowiak D. (2021). COVID-19 Passport as a Factor Determining the Success of National Vaccination Campaigns: Does It Work? The Case of Lithuania vs. Poland. Vaccines.

[25] Szmyd B., Karuga F.F., Bartoszek A., Staniecka K., Siwecka N., Bartoszek A., Błaszczyk M., Radek M. (2021). Attitude and Behaviors towards SARS-CoV-2 Vaccination among Healthcare Workers: A Cross-Sectional Study from Poland. Vaccines.

[26] Szmyd B., Bartoszek A., Karuga F.F., Staniecka K., Błaszczyk M., Radek M. (2021). Medical Students and SARS-CoV-2 Vaccination: Attitude and Behaviors. Vaccines.

[27] Walkowiak M.P., Walkowiak D. (2021). Predictors of COVID-19 Vaccination Campaign Success: Lessons Learnt from the Pandemic So Far. A Case Study from Poland. Vaccines.

[28] Raciborski F., Samel-Kowalik P., Gujski M., Pinkas J., Arcimowicz M., Jankowski M. (2021). Factors Associated with a Lack of Willingness to Vaccinate against COVID-19 in Poland: A 2021 Nationwide Cross-Sectional Survey. Vaccines.

[29] Coyle P.K., Gocke A., Vignos M., Newsome S.D. (2021). Vaccine Considerations for Multiple Sclerosis in the COVID-19 Era. Adv. Ther..

[30] MacDonald N.E. (2015). SAGE Working Group on Vaccine Hesitancy. Vaccine hesitancy: Definition, scope and determinants. Vaccine.

[31] Dubé E., Laberge C., Guay M., Bramadat P., Roy R., Bettinger J. (2013). Vaccine hesitancy: An overview. Hum. Vaccines Immunother..

[32] Gualano M.R., Olivero E., Voglino G., Corezzi M., Rossello P., Vicentini C., Bert F., Siliquini R. (2019). Knowledge, attitudes and beliefs towards compulsory vaccination: A systematic review. Hum. Vaccines Immunother..

[33] Kurtzke J.F. (1983). Rating neurologic impairment in multiple sclerosis: An expanded disability status scale (EDSS). Neurology.

[34] Boguszewski R., Makowska M., Podkowinska M. (2021). A Typology of Poles’ Attitudes toward COVID-19 during the First Wave of the Pandemic. Int. J. Environ. Res. Public Health.

[35] Khataee H., Scheuring I., Czirok A., Neufeld Z. (2021). Effects of social distancing on the spreading of COVID-19 inferred from mobile phone data. Sci. Rep..

[36] Brennen J.S., Simon F.M., Howard P.N., Nielsen R.K. (2020). Types, Sources, and Claims of COVID-19 Misinformation. Ph.D. Thesis.

[37] Sahraian M.A., Gheini M.R., Rezaeimanesh N., Ghajarzadeh M., Naser Moghadasi A. (2020). Knowledge regarding COVID-19 pandemic in patients with Multiple sclerosis (MS): A report from Iran. Mult. Scler. Relat. Disord..

[38] Sulistyawati S., Rokhmayanti R., Aji B., Wijayanti S.P.M., Hastuti S.K.W., Sukesi T.W., Mulasari S.A. (2021). Knowledge, Attitudes, Practices and Information Needs During the COVID-19 Pandemic in Indonesia. Risk Manag. Healthc Policy.

[39] National Multiple Sclerosis Society COVID-19 Vaccine Guidance for People Living with MS. https://www.nationalmssociety.org/coronavirus-covid-19-information/multiple-sclerosis-and-coronavirus/covid-19-vaccine-guidance.

[40] World Health Organization COVID-19 Vaccines. https://www.who.int/emergencies/diseases/novel-coronavirus-2019/covid-19-vaccines.

[41] Centers for Disease Control and Prevention Science Brief: COVID-19 Vaccines and Vaccination. https://www.cdc.gov/coronavirus/2019-ncov/science/science-briefs/fully-vaccinated-people.html.

[42] NAWA Polish National Agency for Academic Exchange Vaccination in Poland: How to Apply and Other Things You Need to Know. https://study.gov.pl/news/vaccination-poland.

[43] Szczepienia Przeciw COVID-19 u Chorych Na Stwardnienie Rozsiane. Stanowisko Polskiego Towarzystwa Neurologicznego. https://szczepienia.pzh.gov.pl/szczepienia-przeciw-covid-19-chorych-na-stwardnienie-rozsiane-stanowisko-polskiego-towarzystwa-neurologicznego/.

[44] Nojszewska M., Kalinowska A., Adamczyk-Sowa M., Kułakowska A., Bartosik-Psujek H. (2021). COVID-19 mRNA vaccines (Pfizer-BioNTech and Moderna) in patients with multiple sclerosis: A statement by a working group convened by the Section of Multiple Sclerosis and Neuroimmunology of the Polish Neurological Society. Neurol. Neurochir. Pol..

[45] Raciborski F., Jankowski M., Gujski M., Pinkas J., Samel-Kowalik P. (2021). Changes in Attitudes towards the COVID-19 Vaccine and the Willingness to Get Vaccinated among Adults in Poland: Analysis of Serial, Cross-Sectional, Representative Surveys, January-April 2021. Vaccines.

[46] Serrazina F., Sobral Pinho A., Cabral G., Salavisa M., Correia A.S. (2021). Willingness to be vaccinated against COVID-19: An exploratory online survey in a Portuguese cohort of multiple sclerosis patients. Mult. Scler. Relat. Disord..

[47] Ehde D.M., Roberts M.K., Herring T.E., Alschuler K.N. (2021). Willingness to obtain COVID-19 vaccination in adults with multiple sclerosis in the United States. Mult. Scler. Relat. Disord..

[48] Rzymski P., Zeyland J., Poniedziałek B., Małecka I., Wysocki J. (2021). The Perception and Attitudes toward COVID-19 Vaccines: A Cross-Sectional Study in Poland. Vaccines.

[49] Ehde D.M., Roberts M.K., Humbert A.T., Herring T.E., Alschuler K.N. (2021). COVID-19 vaccine hesitancy in adults with multiple sclerosis in the United States: A follow up survey during the initial vaccine rollout in 2021. Mult. Scler. Relat. Disord..

[50] Zhang Y., Staker E., Cutter G., Krieger S., Miller A.E. (2021). Perceptions of risk and adherence to care in MS patients during the COVID-19 pandemic: A cross-sectional study. Mult. Scler. Relat. Disord..

[51] Louapre C., Collongues N., Stankoff B., Giannesini C., Papeix C., Bensa C., Deschamps R., Créange A., Wahab A., Pelletier J. (2020). Clinical Characteristics and Outcomes in Patients With Coronavirus Disease 2019 and Multiple Sclerosis. JAMA Neurol..

[52] Capasso N., Palladino R., Montella E., Pennino F., Lanzillo R., Carotenuto A., Petracca M., Iodice R., Iovino A., Aruta F. (2020). Prevalence of SARS-CoV-2 Antibodies in Multiple Sclerosis: The Hidden Part of the Iceberg. J. Clin. Med..

[53] Sepúlveda M., Llufriu S., Martínez-Hernández E., Català M., Artola M., Hernando A., Montejo C., Pulido-Valdeolivas I., Martínez-Heras E., Guasp M. (2021). Incidence and Impact of COVID-19 in MS: A Survey From a Barcelona MS Unit. Neurol. Neuroimmunol. Neuroinflammation.

[54] Czarnowska A., Brola W., Zajkowska O., Rusek S., Adamczyk-Sowa M., Kubicka-Bączyk K., Kalinowska-Łyszczarz A., Kania K., Słowik A., Wnuk M. (2021). Clinical course and outcome of SARS-CoV-2 infection in multiple sclerosis patients treated with disease-modifying therapies—the Polish experience. Neurol. Neurochir. Pol..

[55] Raport Zakażeń Koronawirusem (SARS-CoV-2)—Koronawirus: Informacje I Zalecen. https://www.gov.pl/web/koronawirus/wykaz-zarazen--koronawirusem-sars-cov-2.

[56] Moss B.P., Mahajan K.R., Bermel R.A., Hellisz K., Hua L.H., Hudec T., Husak S., McGinley M.P., Ontaneda D., Wang Z. (2020). Multiple sclerosis management during the COVID-19 pandemic. Mult. Scler. J..

[57] Palmer K., Monaco A., Kivipelto M., Onder G., Maggi S., Michel J.P., Prieto R., Sykara G., Donde S. (2020). The potential long-term impact of the COVID-19 outbreak on patients with non- communicable diseases in Europe: Consequences for healthy ageing. Aging Clin. Exp. Res..

[58] Brownlee W., Bourdette D., Broadley S., Killestein J., Ciccarelli O. (2020). Treating multiple sclerosis and neuromyelitis optica spectrum disorder during the COVID-19 pandemic. Neurology.

[59] Giovannoni G., Hawkes C., Lechner-Scott J., Levy M., Waubant E., Gold J. (2020). The COVID-19 pandemic and the use of MS disease modifying therapies. Mult. Scler. Relat. Disord..

[60] Vogel A.C., Schmidt H., Loud S., Mcburney R., Mateen F.J. (2020). Impact of the COVID-19 pandemic on the health care of >1,000 People living with multiple sclerosis: A cross-sectional study. Mult. Scler. Relat. Disord..

